# Integration of volatilomics, non-targeted metabolomics, and electronic sensory technologies reveals the flavor evolution mechanism in ‘Queen Nina’ table grapes during ripening

**DOI:** 10.1016/j.fochx.2026.104436

**Published:** 2026-09-12

**Authors:** Qiuping Li, Zilin Wang, Bo Zhang, Yameng Liu, Min Yang, Xinyi Huang

**Affiliations:** a School of Ethnic Medicine, Yunnan Minzu University, Kunming 650504, China; b College of Agronomy and Biotechnology, Yunnan Agricultural University, Kunming 650201, China; c College of Food Science and Technology, Yunnan Agricultural University, Kunming 650201, China

**Keywords:** Table grapes, Fruit flavor, Metabolic pathway, Electronic sensory analysis, Aroma compounds

## Abstract

‘Queen Nina’ grapes are renowned for their distinctive “fruity”, “cheesy”, and “vinous” aromas, yet the metabolic mechanisms driving these traits during ripening remain elusive. This study integrated volatile metabolomic, non targeted metabolomic, and electronic sensory technologies to profile flavor evolution across three developmental stage. We identified 44 core differential volatile organic compounds (VOCs) closely associated with the transition of profiles. Specifically, (Z)-3-hexen-1-ol declined, while esters including (E)-2-hexen-1-ol acetate and ethyl 3-hydroxybutanoate accumulated. ROAV analysis identified ethyl butanoate and hexanoate as key aroma-active compounds. Furthermore, the downregulation of 355 non-volatile metabolites (amino acids, lipids) may serve as potential biosynthetic precursors for VOCs, whereas upregulated flavonoids are associated with enhanced coloration. A constructed metabolic pathway map revealed how primary metabolism fuels secondary pathways to orchestrate flavor development. These findings elucidate the metabolic basis of the distinctive aroma characteristics of ‘Queen Nina’ grapes and provide theoretical insights for further investigation into the quality characteristics and formation mechanisms in table grapes.

## Highlights

• Multi-omics revealed flavor evolution in ‘Queen Nina’ grapes.
• Esters drive the shift from “grassy” to “fruity-cheesy” aroma.
• Ethyl butanoate and hexanoate are key “cheesy” aroma markers.
• Lipid and amino acid degradation fuel volatile biosynthesis.
• A metabolic map elucidates the synergy of primary-secondary pathways.

## Graphical abstract

**Figure:**
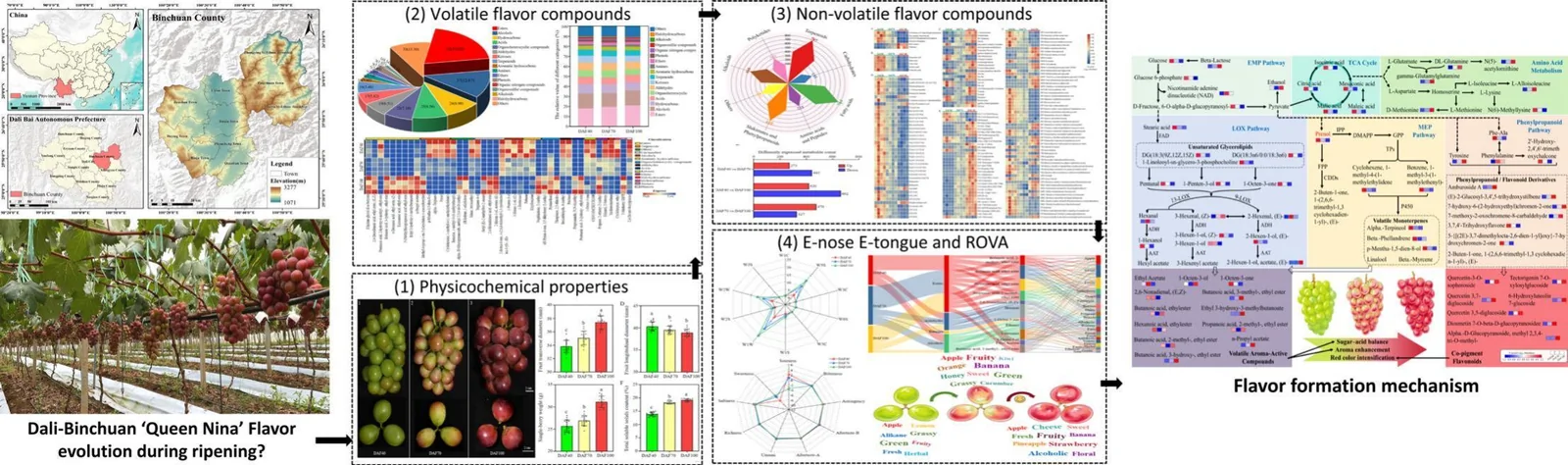

## 1 Introduction

Grapes (*Vitis vinifera* L.) are among the most economically significant fruit crops globally. Table grapes, specifically, refer to varieties intended for fresh consumption rather than for winemaking, drying, or processing. (Yue et al., 2020; Wang, Yan, et al., 2025). According to data from the International Organisation of Vine and Wine (OIV), China is the world's largest producer of table grapes. China's grape with an annual grape output of about 13.5 million tons, accounting for 42% of global table grape production (Wang, Yan, et al., 2025).The quality evaluation of table grapes requires the comprehensive consideration of multiple interrelated indicators. However, fundamental sensory attributes, including appearance, texture, and aroma, are assigned the highest priority in determining consumer preference and market value (Wang et al., 2023; Zheng et al., 2025).

The aroma profile of table grapes is determined by a complex array of VOCs. Over 500 VOCs have been identified across different grape varieties, including terpenoids, esters, norisoprenoids, alcohols, aldehydes, and ketones. The biosynthesis of these aromatic compounds involves multiple metabolic pathways that interact synergistically throughout fruit development (Martínez et al., 2025). The lipoxygenase (LOX) pathway plays a key role in generating C6 aldehydes and alcohols from polyunsaturated fatty acids, thereby contributing to the characteristic “green grass” or “green leaf” aroma of immature berries (Jin et al., 2025). Esters primarily contribute “fruity” and “sweet” aromas (Qian et al., 2016). Notably, certain non-volatile compounds, such as free amino acids, not only directly contribute to taste perception by exhibiting bitter, sweet, umami, or aromatic characteristics, but also serve as important precursors for the biosynthesis of volatile aroma compounds (Liao et al., 2022).

‘Queen Nina’, a tetraploid table grape cultivar of *Vitis labrusca* L. × *Vitis vinifera* L. origin, was developed through the cross between the cultivars ‘Akitsu-20’ and ‘Aki-Queen’ by the National Institute of Fruit Tree Science (NIFTS), Japan (Sato et al., 2013). It has captured widespread attention in the Chinese market and international premium consumption sectors, owing to its bright red coloration, large berries, and distinctive flavor characterized by subtle notes of “fruity”, “cream”, and “red wine” (Wang, Zhang, et al., 2025). Temperature fluctuation is a key physiological driver for optimizing carbohydrate metabolism in perennial crops (Chen, Mkunga, et al., 2025). Binchuan County, as the primary cultivation region for ‘Queen Nina’ grapes in Yunnan Province, China, is characterized by its unique geographical location and climatic conditions, which result in pronounced diurnal temperature variations. These conditions greatly promote the biosynthesis and accumulation of the characteristic aroma-related metabolites in ‘Queen Nina’ grapes. Previous studies have primarily focused on the aroma components of ‘Queen Nina’ with particular emphasis on esters as the key determinants of its typical flavor (Wang, Zhang, et al., 2025). However, the synergistic contribution of VOCs，and non-volatile substances to the overall flavor and color perception of the ‘Queen Nina’ cultivar remains relatively underexplored. This situation has resulted in insufficient in-depth exploration of the differential compounds and the regulatory mechanisms among metabolic pathways involved in the formation of characteristic flavor and aroma during its ripening process.

Therefore, this study employed a combined approach of metabolomics and electronic sensory technologies to comparatively analyze the dynamic changes in flavor during the ripening of ‘Queen Nina’ grapes cultivated in Binchuan, Dali. By integrating volatile and non-volatile metabolomics data, we aimed to elucidate the metabolic pathways underlying the characteristic aroma of this cultivar and to construct a comprehensive pathway map illustrating its flavor evolution. These findings will provide a theoretical basis for understanding flavor formation in table grapes.

## 2 Materials and methods

### 2.1 Plant materials and sample collection

**Fig. 1:**
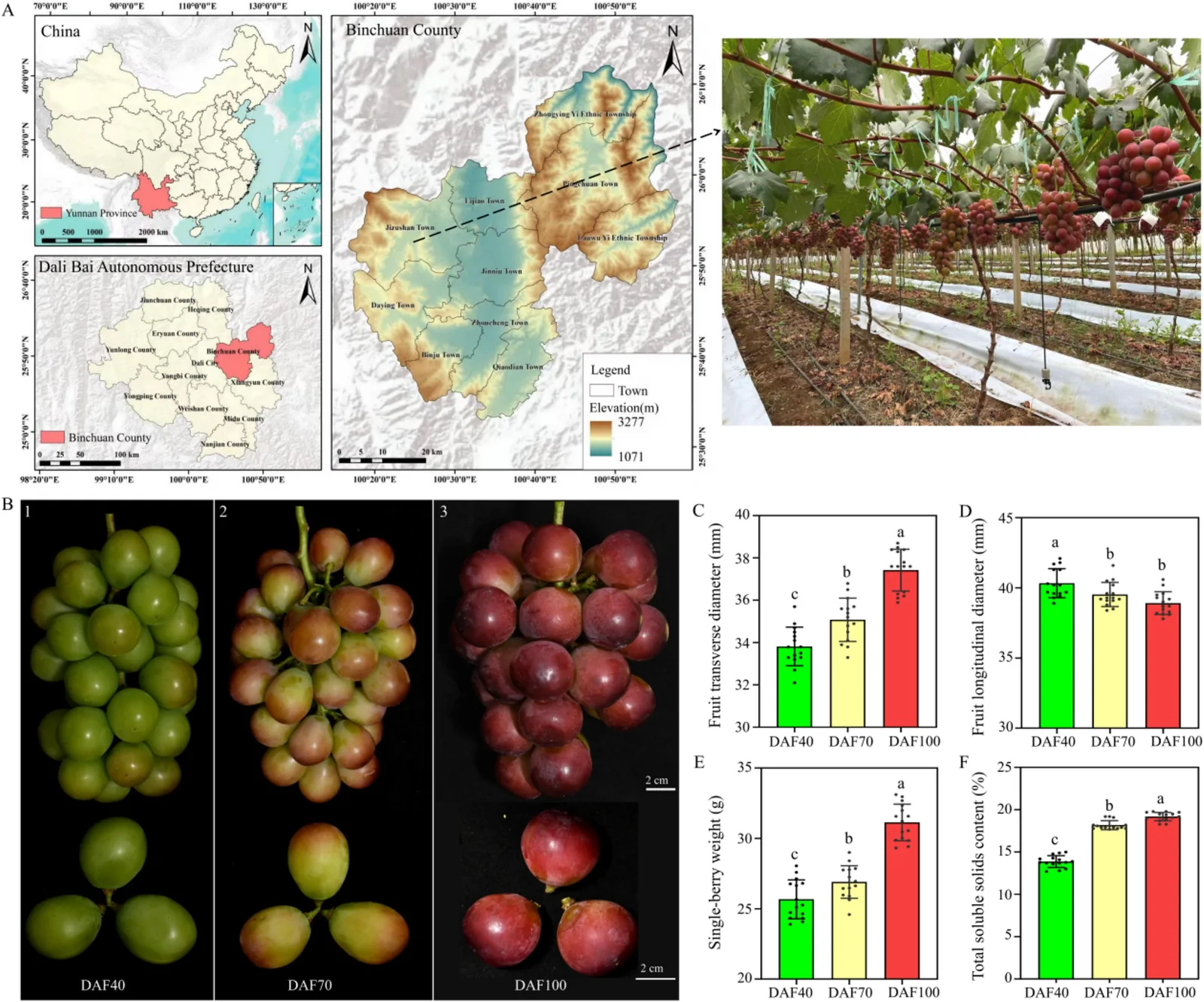
Morphological and physicochemical changes during the development of ‘Queen Nina’ grapes. (A) Map indicating the experimental vineyard located in Jizushan Town, Binchuan County, Dali Prefecture, Yunnan Province, China (left), and a representative photograph of the grape orchard (right). (B) Appearance of ‘Queen Nina’ grape clusters (upper panels) and individual berries (lower panels) at three developmental stages: DAF40, DAF70, and DAF100. (C–F) Physicochemical properties of ‘Queen Nina’ berries at DAF40, DAF70, and DAF100. (C) Transverse diameter, (D) Longitudinal diameter, (E) Single berry weight, and (F) Total soluble solids content. Data are presented as mean ± SD (*n* = 15). Statistical significance was determined by one-way ANOVA; different lowercase letters above the bars indicate significant differences between groups (*P* < 0.05).

‘Queen Nina’ grape berries were collected from a commercial orchard located in Binchuan County, Dali Bai Autonomous Prefecture, Yunnan Province, China (approximately 25.8°N, 100.4°E; altitude: 1810 m) during the 2025 growing season (June to August) (Fig. 1A). All grapevines were established by grafting ‘Queen Nina’ scions onto a uniform rootstock ‘3309 M’, and had been growing for six years post-grafting. The vines were trained to a double-cross V-shaped trellis system and managed under identical cultivation conditions, including irrigation, fertilization, and pruning practices.

Berries were harvested at three developmental stage based on days after flowering (DAF): Stage I (40 days after flowering, DAF40), Stage II (70 days after flowering, DAF70), and Stage III (100 days after flowering, DAF100). The DAF100 time point was designated as the commercial maturity stage based on local cultivation practice, as growers in the Binchuan region typically harvest ‘Queen Nina’ at approximately 100 days after flowering. At each stage, approximately total 3 kg of healthy and uniform-sized berries were randomly collected from five vines as a pool, with each pool serving as one biological replicate. Samples were immediately transported to the laboratory on ice. For each biological replicate, fifteen berries were randomly selected for measurement of physical parameters (berry weight, longitudinal and transverse diameters, and total soluble solids). The remaining berries were deseeded, frozen in liquid nitrogen, and stored at −80 °C for subsequent analyses of total sugar, titratable acidity, volatile organic compounds, and widely targeted metabolomics.

### 2.2 Physical characteristics and basic quality parameters

For each developmental stage, fifteen uniformly sized grape berries were randomly selected from each biological replicate to determine physical traits and soluble solids content. Single berry weight was measured using an electronic analytical balance (ME204E, Mettler-Toledo, Switzerland, readability:0.0001 g). Longitudinal and transverse diameters were determined using a digital Vernier caliper (0–150 mm, 0.01 mm precision, Shanghai STWC, China). Total soluble solids content was measured from fresh juice using a digital refractometer (PAL-1, Atago, Japan range: 0.0–53.0% Brix) at room temperature (25 ± 1 °C). The refractometer was calibrated with distilled water before each measurement session (Chen, Wen, et al., 2025).

### 2.3 Total sugar content

Total sugar content was determined using a commercial assay kit (JC0409-M, Nanjing Jice Biotechnology, China) based on the 3,5-dinitrosalicylic acid (DNS) method. Briefly, 0.1 g of homogenized pulp was extracted with 1 mL of the provided acid reagent (hydrochloric acid solution) and 1.5 mL of distilled water, and then hydrolyzed at 95 °C for 30 min. After centrifugation, the supernatant was collected and reacted with DNS reagent at 95 °C for 10 min. The absorbance was measured at 540 nm using a microplate reader (Infinite 200 PRO, TECAN, Switzerland). Total sugar content was calculated from a glucose standard curve and expressed as mg/g fresh weight (FW). Each sample assay consisted of three replicates.

### 2.4 Titratable acidity

Titratable acidity was determined by acid-base titration according to the Chinese National Standard GB 12456–2021 with minor modifications. Fresh grape sample (5.0 g) was homogenized with 10 mL of distilled water and heated in a boiling water bath for 30 min with occasional shaking. After cooling, the volume was adjusted to 25 mL with boiled distilled water and filtered. An aliquot of 10 mL of the filtrate was titrated with 0.1 N NaOH standard solution using 1% phenolphthalein as an indicator until a persistent pink color appeared for 30 s. A blank titration was performed with distilled water. Titratable acidity was calculated using the formula:

$$X=\frac{c\times \left(V1-V2\right)\times K\times F}{m}\times 1000$$

where c is the NaOH concentration (mol/L), V1 and V2 are the titration volumes for sample and blank (mL), K is the conversion factor for citric acid (0.070), F is the dilution factor, and m is the sample mass (g). Results were expressed as g/kg fresh weight. Each biological replicate was analyzed once.

### 2.5 Electronic nose analysis

The volatile fingerprint of ‘Queen Nina’ grape berries at different developmental stage was analyzed using a portable electronic nose system (PEN3, Airsense Analytics GmbH, Germany) following previously described methods with slight modifications (Liu et al., 2025). Ten grape berries were randomly selected from each sample, deseeded, and immediately ground into a fine powder under liquid nitrogen. An aliquot of 5.0 g of the powder was placed into a 40 mL headspace vial, sealed with parafilm, and equilibrated at room temperature (25 ± 1 °C) for 30 min to enrich the headspace volatiles. The *E*-nose was equipped with 10 metal oxide sensors corresponding to different sensitive compounds: W1C (aromatic compounds), W5S (broad-range, sensitive to nitrogen oxides), W3C (aromatic compounds), W6S (hydrogen), W5C (aromatic-aliphatic compounds), W1S (broad-methane), W1W (sulfur-organic compounds), W2S (broad-alcohol), W2W (sulfur‑chlorine compounds), and W3S (methane-aliphatic compounds) (Liu et al., 2025). The measurement parameters were set as follows: carrier gas flow rate of 300 mL/min, sampling time of 60 s, and cleaning time of 90 s. The stable response values from 50 to 55 s were selected for subsequent analysis. Sensor responses were expressed as the ratio of conductance (G/G₀), where G is the conductivity of the sensor in the sample gas and G₀ is the conductivity in clean air. Each sample assay consisted of three replicates.

### 2.6 Electronic tongue analysis

Taste characteristics of ‘Queen Nina’ grape berries at different developmental stage were evaluated using an electronic tongue system (SA402B, Insert Co., Japan), following the method described by Chen et al. (2023) with minor modifications. Fresh grape berries were deseeded and homogenized. The resulting pulp was filtered through four layers of gauze to remove solid debris, and 40 mL of the clarified juice was collected in a dedicated *E*-tongue sample cup and allowed to equilibrate at room temperature for 10 min prior to analysis.

The E-tongue was equipped with six lipid membrane sensors corresponding to different taste attributes: CA0 (sourness), C00 (bitterness), AE1 (astringency), AAE (umami), CT0 (saltiness), and GL1 (sweetness), along with an Ag/AgCl reference electrode. Sensors were activated and calibrated according to the manufacturer's instructions before each measurement session. The acquisition time for each sample was 120 s, and sensors were automatically rinsed for 30 s between measurements to prevent carry-over effects. Stable response values recorded from 110 to 120 s were averaged and used as the representative output. All data were normalized against a reference solution containing 30 mmol/L KCl and 0.3 mmol/L tartaric acid. Each sample assay consisted of three replicates.

### 2.7 Untargeted metabolomic analysis

Untargeted metabolomic analysis of ‘Queen Nina’ grape berries was conducted following previously described methods (Chen et al., 2013; Sawada et al., 2009). Freeze-dried samples were ground (60 Hz, 30 s), and 25 mg of powder was extracted with 1000 μL of methanol: water (4,1, *v*/v) containing internal standards. The mixture was vortexed, homogenized (45 Hz, 4 min), and sonicated in an ice-water bath for 5 min; this process was repeated three times. After incubation at −40 °C for 1 h, samples were centrifuged at 12,000 rpm for 15 min at 4 °C. The supernatant was filtered (0.22 μm) for LC-MS analysis. A pooled quality control (QC) sample was prepared by mixing equal aliquots of each sample.

Chromatographic separation was performed using a Vanquish UHPLC system (Thermo Fisher Scientific, USA) equipped with a Kinetex C18 column (2.1 mm × 50 mm, 2.6 μm). The mobile phase consisted of (A) 0.01% acetic acid in water and (B) 50% acetonitrile/isopropanol. The flow rate was 0.3 mL/min, column temperature 25 °C, and injection volume 2 μL.

Mass spectrometry was conducted using a Stellar quadrupole linear ion trap mass spectrometer (Thermo Fisher Scientific, USA) with a heated electrospray ionization source operating in parallel reaction monitoring (PRM) mode. Ion source parameters were: spray voltage, 3.8 kV (positive) and − 3.4 kV (negative); sheath gas, 50 Arb; auxiliary gas, 15 Arb; ion transfer tube temperature, 320 °C; vaporizer temperature, 450 °C.

Data acquisition and processing were performed using Biobud software. Metabolites were identified by matching MS/MS spectra with the Biotree plant database (Biotree Biotech, China.). The most stable and specific fragment ion was selected for quantification. QC samples were analyzed throughout the run to monitor instrument stability. The retention time drift of internal standards was within ±12 s, and the relative standard deviation (RSD) of internal standard responses in QC samples ranged from 1.26% to 3.14%, indicating excellent instrument stability and data reliability. Each sample assay consisted of four replicates.

### 2.8 Analysis of volatile organic compounds by HS-SPME-GC–MS

Volatile organic compounds (VOCs) in ‘Queen Nina’ grape berries were analyzed using headspace solid-phase microextraction coupled with gas chromatography–mass spectrometry (HS-SPME-GC–MS) following previously described methods (Liu et al., 2025). Samples were ground into powder under liquid nitrogen. An aliquot of 500 ± 10 mg was placed into a 20 mL headspace vial, and 1 mL of saturated sodium chloride solution containing the internal standard (2-octanol, 10 mg/L) was added. For retention index calculation, 10 μL of n-alkane mixture (C9–C23) was analyzed separately.

A conditioned SPME fiber (50/30 μm DVB/CAR/PDMS, 2 cm, Supelco, USA) was used for extraction. Samples were equilibrated at 60 °C for 15 min, followed by extraction at the same temperature for 30 min. After extraction, the fiber was desorbed at 250 °C for 4 min in splitless mode.

GC–MS analysis was performed using an Agilent 7890B gas chromatograph coupled with an Agilent 5977B mass spectrometer (Agilent Technologies, USA). Volatile compounds were separated on a DB-Wax column (30 m × 250 μm × 0.25 μm). Helium (99.999%) was used as carrier gas at a flow rate of 1.0 mL/min. The oven temperature was programmed as follows: 40 °C held for 4 min, increased to 245 °C at 5 °C/min, and held for 5 min. The temperatures of the inlet, transfer line, ion source, and quadrupole were set at 250 °C, 250 °C, 230 °C, and 150 °C, respectively. Mass spectra were acquired in electron ionization mode (70 eV) over a mass range of *m*/*z* 20–400.

Data were processed using Chroma TOF software (V 4.3×, LECO, USA) and the NIST library for peak extraction, deconvolution, and alignment (Kind et al., 2009). Compounds were identified by matching mass spectra (≥80% match) and retention indices (RI) calculated from n-alkanes (C9–C23). Semi-quantification was performed using peak areas relative to the internal standard. Each sample assay consisted of four replicates.

The contribution of each volatile compound to the overall aroma profile was evaluated by calculating the relative odor activity value (ROAV) according to the method described by Zhang et al. (2020). The ROAV was calculated using the following equation: ROAV_i_ = 100 × (C_i_/C_max_) × (T_max_/T_i_). where C_i_ represents the relative concentration of compound i (expressed as the percentage of its peak area relative to the total peak area of all identified compounds), C_max_ is the relative concentration of the compound with the maximum odor activity value, T_i_ is the odor threshold of compound i, and T_max_ is the odor threshold of the compound with the maximum odor activity value.

The compound with the highest OAV was assigned a reference ROAV of 100. Odor thresholds were obtained from publicly available databases, including FlavorDB2 (https://cosylab.iiitd.edu.in/flavordb2/), PubChem (https://pubchem.ncbi.nlm.nih.gov), the LRI Odor database (https://www.odour.org.uk), and the BFU Food Flavor Database 2.0 (http://foodflavorlab.cn/#/v2/home).

### 2.9 Statistical analysis

Data were compiled using Microsoft Excel 2019 (Microsoft Corporation, Redmond, WA, USA) and statistically analyzed using IBM SPSS Statistics 26 (IBM Corp., Armonk, NY, USA). Differences among groups were evaluated by one-way analysis of variance (ANOVA) followed by Tukey's honestly significant difference (HSD) test, with statistical significance defined at *P* < 0.05. All figures were generated using GraphPad Prism 8.0 (GraphPad Software, San Diego, CA, USA) and refined with Adobe Illustrator 2021 (Adobe Inc., San Jose, CA, USA). Heatmaps were constructed using TBtools software (version 2.304) (Chen et al., 2020). Multivariate analyses, including principal component analysis (PCA) and orthogonal partial least squares discriminant analysis (OPLS-DA), were performed using SIMCA 14.1 software (Umetrics AB, Umeå, Sweden).

## 3 Results and discussion

### 3.1 Observation of grape characteristics at different berry development stages

Grape berry development follows a double-sigmoid growth pattern, comprising three main stages: two rapid growth phases (Stage I and Stage III) separated by a slow-growth phase (Stage II) (Coombe, 1976; Deluc et al., 2007; Yue et al., 2020). During Stage I, the fruit undergoes rapid cell division, accompanied by the accumulation of organic acids. In Stage II, despite its slow growth rate, this intermediate period is characterized by profound physiological and biochemical activities, including embryo development and preparation for véraison. During Stage III, the fruit enters the second rapid growth phase, marked by further cell expansion, continued accumulation of color and aroma compounds, and peak sugar accumulation. Therefore, a systematic understanding of flavor dynamics in table grapes during these critical developmental stages, along with the identification of reliable biomarkers, is essential for ensuring fruit quality.

As described above, ‘Queen Nina’ grapes were compared and analyzed across three representative stages—namely, the DAF40 (Stage I), DAF70 (Stage II), and DAF100 (Stage III) (Fig. 1A). ‘Queen Nina’ at the DAF40 displayed a sour and astringent taste with firm flesh, whereas mature berries developed a rich, mellow flavor with a sweet aftertaste, tender and juicy pulp, and a persistent “wine” aroma accompanied by “cheese” aroma (Fig. 1B). We measured the transverse and longitudinal diameters, single fruit weight, and sugar content of grape berries at different developmental stages. As shown in Fig. 1C and Fig. 1D, the transverse diameter and shape index reflected the morphological characteristics of the grapes. The transverse diameter gradually increased throughout the growth period, whereas the longitudinal diameter exhibited a decreasing trend. This pattern corresponds to the progressive morphological transition of the berries from an elongated to an oval shape during maturation. Moreover, the individual fruit weight of ‘Queen Nina’ gradually increased during the ripening process (Fig. 1E), which may be attributed to both the progressive accumulation of water and the concurrent increase in soluble solids content (Wang, Zhang, et al., 2025). For instance, the increase in total soluble solids from 13.83% to 19.26% (Fig. 1F).

### 3.2 Energy metabolism substances at different maturities

The physiological changes during grape growth encompass not only physical attributes but also the composition of sugars and organic acids, which play a crucial role in determining the taste of table grapes (Liu et al., 2006). As shown in Table 1, the titratable acidity content gradually decreased during the development of ‘Queen Nina’, whereas the total sugar content increased from 87.38 mg/g to 141.63 mg/g. This trend contributed to the sweet taste of the grapes at the DAF100.

**Table 1:** Changes in sugar and organic acid contents in ‘Queen Nina’ grapes at different developmental stage.

Parameter	DAF40	DAF70	DAF100
Total sugar (mg/g)	87.38 ± 2.33 ^c^	109.48 ± 2.38 ^b^	141.63 ± 6.69 ^a^
Titratable acid (g/kg)	9.22 ± 0.010 ^a^	7.29 ± 0.007 ^b^	3.99 ± 0.004 ^c^
Fructose (nmol/g)	1,309,560.00 ± 43,525.60 ^b^	1,401,090.00 ± 79,069.84 ^b^	1,552,983.33 ± 31,113.72 ^a^
d-Glucose (nmol/g)	2,482,780.00 ± 36,053.21 ^a^	2,354,180.00 ± 124,823.75 ^ab^	2,190,510.00 ± 66,049.39 ^b^
Malic acid (nmol/g)	32,197.07 ± 870.53 ^a^	22,589.03 ± 764.41 ^b^	14,919.73 ± 281.11 ^c^
Citric acid (nmol/g)	8131.07 ± 34.38 ^a^	4788.33 ± 188.40 ^b^	3960.86 ± 69.53 ^c^

Data are presented as mean ± SD (*n* = 3). Statistical significance was determined by one-way ANOVA. Within each stage, different lowercase letters indicate significant differences between groups at *P* < 0.05.

In this study, metabolomic profiling revealed that the predominant organic acid in the berries was malic acid, accompanied by a small amount of citric acid, while tartaric acid was not detected (Table 1). This result is contrary to that reported by Wang, Zhang, et al. (2025) who found abundant tartaric acid in ‘Queen Nina’. This discrepancy may be attributed to differences in the geographical cultivation regions. The content of these organic acids gradually decreased with grape development. Furthermore, Wei et al. (2023) pointed out that maintaining a relatively low ratio of tartaric acid to malic acid is crucial for table grape varieties to ensure optimal fruit flavor. The primary sugars in ‘Queen Nina’ are glucose and fructose. These monosaccharides not only contribute to sweetness but also act as precursors for aromatic compounds (Kunter et al., 2024).

Glucose content slightly decreased during the DAF100 of the berries, whereas fructose content showed a slight increase (Table 1). Given that fructose possesses a relative sweetness approximately 1.75 times that of sucrose, compared to only 0.75 times for glucose, the shift in hexose composition significantly enhances the perceived sweetness of the fruit (Shammai et al., 2018).

### 3.3 Profiling and compositional changes of volatile organic compounds (VOCs)

**Fig. 2:**
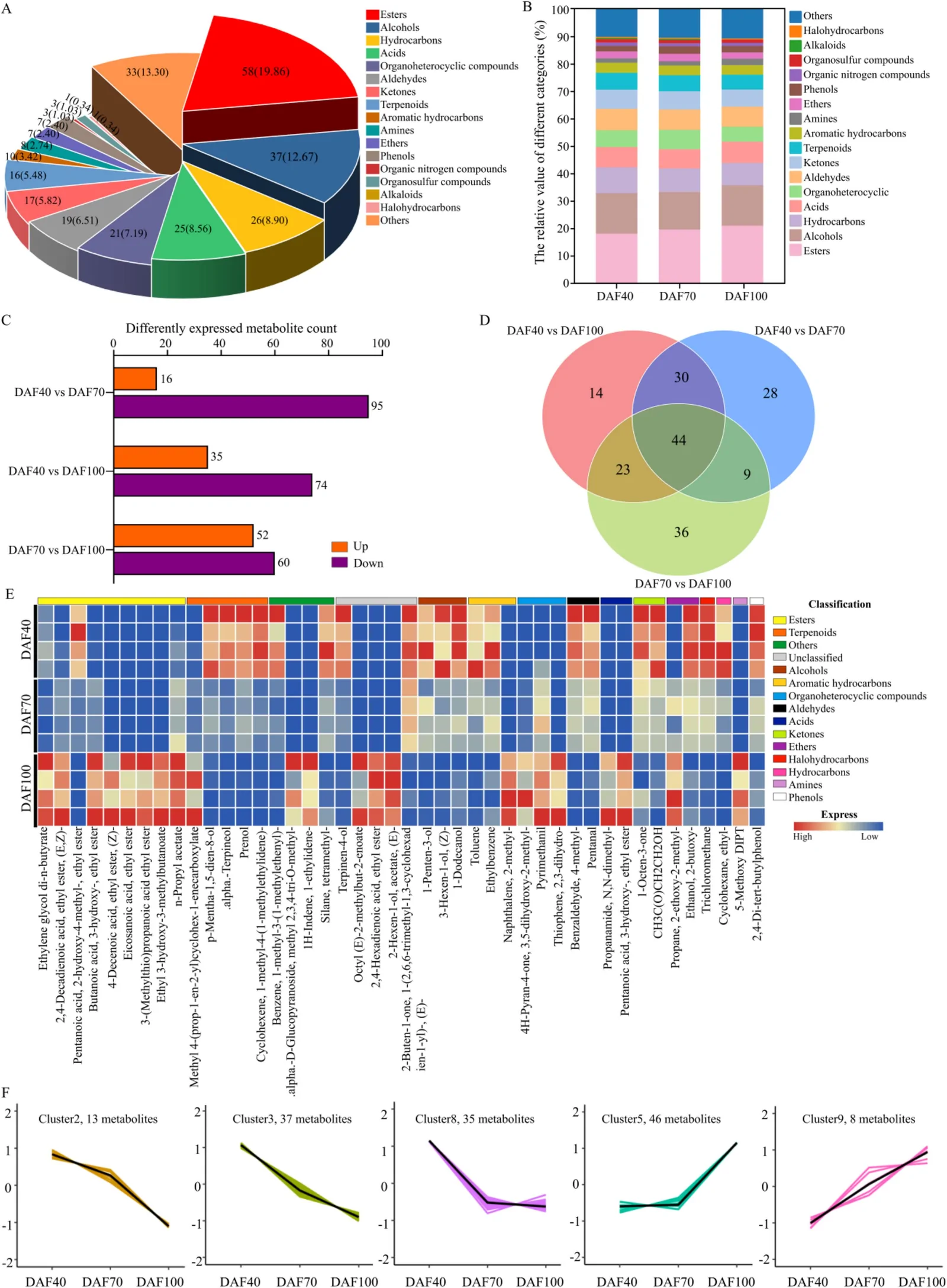
Profiling and dynamic changes of volatile organic compounds (VOCs) in ‘Queen Nina’ grapes (DAF40, DAF70, and DAF100). (A) 3D pie chart showing the compositional classes and proportions of total VOCs. (B) Stacked bar chart showing the distribution of VOC classes at each developmental stage. (C) Numbers of differentially accumulated VOCs in pairwise comparisons. (D) Venn diagram displaying the overlap of differential VOCs among the three comparisons. (E) Heatmap visualization of the 44 key differential VOCs. The color scale represents normalized abundance, with red indicating high abundance and blue indicating low abundance. (F) Representative K-means clustering patterns of the 44 differential VOCs. (For interpretation of the references to color in this figure legend, the reader is referred to the web version of this article.)

A total of 292 VOCs with CAS registry numbers were identified in this study. These comprised 58 esters, 37 alcohols, 26 hydrocarbons, 25 acids, 21 organic heterocyclic compounds, 19 aldehydes, 17 ketones, 16 terpenes, 10 aromatic hydrocarbons, 8 amines, 14 ethers and phenols, 6 organic nitrogen compounds and organic sulfur compounds, 2 alkaloids and halohydrocarbons, and 33 other compounds (Fig. 2A; Table S1). The classes of these volatile compounds were similar to those observed in most grape varieties during growth (Li et al., 2023; Villano et al., 2023). Among these, esters, alcohols, and hydrocarbons were the predominant volatile compounds throughout the growth process, accounting for 19.86%, 12.67%, and 8.90% of the total VOCs, respectively (Fig. 2B).

These compound classes are largely derived from the fatty acid oxidation pathway, in which linolenic and linoleic acids are sequentially converted to C6 aldehydes and alcohols via LOX, hydroperoxide lyase (HPL) and alcohol dehydrogenase (ADH). In wine grape varieties such as ‘Cabernet Sauvignon’, volatiles from this pathway particularly (E)-2-hexenal, hexanol, and (Z)-3-hexen-1-ol can represent 15–25% of total VOCs and are primarily responsible for ‘green’ and ‘grassy’ aromas (Qian et al., 2016). In ‘Queen Nina’, the same compound classes were present but showed distinct accumulation patterns: the abundance of LOX-derived volatiles declined progressively during ripening (Cluster 3, Fig. 2F), while esters, particularly those derived from fatty acid esterification, became increasingly dominant. This shift suggests that the flavor transition from ‘green’ to “fruity” in ‘Queen Nina’ is driven not only by the decline of LOX activity but also by the concurrent activation of ester biosynthesis pathways, a feature that may distinguish this tetraploid table grape from wine grape varieties.

In the present study, aldehydes and alcohols were decreased. This decline coincided with a reduction in the aldehyde-to-alcohol ratio toward maturity. Notably, this pattern contrasts with the increase in alcohol concentrations reported during ripening in ‘Cannonau’ grapes (Petretto et al., 2021), but aligns with the decline observed in ‘Pinot Noir’ and ‘Shiraz’ following over-ripening (Šuklje et al., 2019; Yuan & Qian, 2016). The similarity to wine grape varieties, despite ‘Queen Nina’ being a table grape, suggests that the metabolic shift from LOX-derived volatiles to ester biosynthesis is a conserved feature of grape ripening.

In ‘Queen Nina’, volatile phenolic compounds were present at low levels and declined progressively during ripening (Fig. 2A). Volatile phenolic compounds in grapes predominantly exist in glycosidically bound forms (Noguerol-Pato et al., 2012), and their levels decline during the DAF100. This pattern is consistent with that observed in ‘Pinot Noir’, where these compounds reach their maximum concentration at ripening, followed by a rapid decrease within one week thereafter (Yuan & Qian, 2016). However, the low abundance of volatile phenolics in ‘Queen Nina’ relative to ‘Pinot Noir’ further supports the conclusion that ester biosynthesis, rather than phenolic compound accumulation, is the primary driver of aroma development in this cultivar.

Taken together, these variations may reflect the complex biochemical pathways underlying the generation of volatile organic compounds during the ripening of ‘Queen Nina’ grapes.

### 3.4 Identification of core differential VOCs and their temporal patterns

Orthogonal partial least squares discriminant analysis (OPLS-DA) was applied to analyze the metabolite profiles of grape samples at different developmental stage, revealing distinct inter-stage metabolic differences (Fig. S1A–C). The model demonstrated high reliability and predictive capacity, with R^2^ values approaching 1, consistent with standard metabolomic studies (Zhang et al., 2025).

Subsequently, Pairwise comparisons (VIP > 1, *P* < 0.05) identified 111 (DAF40 vs. DAF70), 112 (DAF70 vs. DAF100), and 109 (DAF40 vs. DAF100) differential VOCs, with up−/down-regulated ratios of 16/95, 52/60, and 35/74, respectively (Fig. 2C; Fig. S1D–F;). A Venn diagram across the three comparisons yielded 44 common differential VOCs (Table S2; Fig. 2D), comprising esters (9), terpenes (5), alcohols (3), aromatic hydrocarbons (3), aldehydes (2), acids (2), ketones (2), ethers (2), halohydrocarbons (1), and others (Fig. 2E). K-means clustering of their temporal accumulation patterns revealed nine clusters, of which five (Clusters 2, 3, 8, 5, and 9) showed clear stage-specific trends (Fig. 2F):

(1) Cluster 2 contained 2-Buten-1-one, 1-(2,6,6-trimethyl-1,3-cyclohexadien-1-yl)- sharply declined from DAF70 to DAF100. This compound belongs to the C13-norisoprenoids. Typically decreasing during ripening as carotenoid cleavage dioxygenase (CCD) activity declines (Liu et al., 2025), showed a similar pattern to that reported in yellow peach during the “green” to “fruity”aroma transition (Liu et al., 2025). Its decline in ‘Queen Nina’ coincided with the accumulation of fruity esters (Cluster 5), further supporting its role as a marker of the “fruity” shift. Among these, β-ionone exhibits a characteristic “violet” aroma, while β-damascone predominantly emits “apple” flavors. Although C13-norisoprenoids occur at relatively low concentrations, their extremely low odor thresholds enable them to contribute significantly to the overall aroma profile of fruits (Panighel & Flamini, 2014).
(2) Cluster 3 exhibited a continuous decline from DAF40 to DAF100. The VOCs contained trichloromethane, ethanol, 2-butoxy-; 1-dodecanol; CH3C(*O*)CH2CH2OH; 2,4-di-tert-butylphenol; pentanal; benzaldehyde, 4-methyl-; silane, tetramethyl-; prenol; ethylbenzene; 1-octen-3-one; (Z)-3-hexen-1-ol; p-mentha-1,5-dien-8-ol, toluene; cyclohexane, ethyl-; and 1-penten-3-ol. These compounds indicate that cluster 3 was enriched in C6/C5 aldehydes and certain C6 alcohols. Among these, In ‘Queen Nina’, the LOX-derived compounds (Z)-3-hexen-1-ol and pentanal were most abundant at DAF40 and declined thereafter (Cluster 3, Fig. 2F), consistent with the decline in LOX activity during ripening (Qian et al., 2016). However, (Z)-3-Hexen-1-ol and hexanol have also been identified as secondary contributors to the varietal flavor differences among wine grape varieties such as ‘Grenache’, ‘Tempranillo’, ‘Merlot’, and ‘Cabernet Sauvignon’ (Goulioti et al., 2025), suggesting a prolonged retention of “green” aroma that may contribute to flavor complexity. The C6 alcohol-to-aldehyde ratio increased progressively during ripening, confirming the shift from “green” to “fruity” aroma profiles (Goulioti et al., 2025).
(3) Cluster 8 exhibited a sharp decline from DAF40 to DAF70. This dynamic pattern strongly suggests that the compounds in cluster 8 are primarily associated with the physiological and biochemical processes occurring during early fruit development, potentially serving as stress-related metabolites, biosynthetic precursors, or volatile compounds with specific ecological functions. Their rapid decrease marks a definitive metabolic shift in grapes from growth to ripening. This cluster include Cyclohexene, 1-methyl-4-(1-methylethylidene)-; Pentanoic acid, 2-hydroxy-4-methyl-, ethyl ester; Terpinen-4-ol; alpha.-Terpineol; Benzene, 1-methyl-3-(1-methylethenyl)- which exhibited a sharp decline from DAF40 to DAF70 (Fig. 2F). These compounds as aroma precursors in grapes, particularly in aromatic varieties where they contribute “citrus” and “herbal” notes (Yue et al., 2020). However, unlike muscat-type grapes, where monoterpenes accumulate throughout ripening and contribute significantly to the final aroma (Mele et al., 2021), the rapid decline of these compounds in ‘Queen Nina’ suggests that they serve primarily as transient precursors metabolites during early development rather than as major contributors to the mature flavor profile. The relatively high concentrations of terpinen-4-ol and α-terpineol at DAF40 may reflect active oxidative metabolism in immature berries or their glycosylation as aroma precursors (Yue et al., 2020). Their rapid decrease marks a definitive metabolic shift from early developmental processes toward ripening-associated ester biosynthesis in ‘Queen Nina’ (Kalua & Boss, 2009).

However, unlike muscat-type varieties where terpenoids accumulate and contribute intense floral aromas (Mele et al., 2021), the rapid decline in ‘Queen Nina’ suggests a limited role for terpenoids in its mature flavor. In addition, Pentanoic acid, 2-hydroxy-4-methyl-, ethyl ester contributes “fruity” or “cheese” in fermented foods, and its occurrence during early grape development may be associated with amino acid metabolism (Kong et al., 2019).

(1) Cluster 5 contained a total of 18 differential compounds, including 2-Hexen-1-ol, acetate, (*E*)-; 2,4-Hexadienoic acid, ethyl ester; Naphthalene, 2-methyl-; Ethyl 3-hydroxy-3-methylbutanoate; Pentanoic acid, 3-hydroxy-, ethyl ester; 5-Methoxy DIPT; Thiophene, 2,3-dihydro-; 3-(Methylthio)propanoic acid ethyl ester; 1H-Indene, 1-ethylidene-; Eicosanoic acid, ethyl ester; 4-Decenoic acid, ethyl ester, (*Z*)-; Propanamide, *N*,*N*-dimethyl-; alpha.-D-Glucopyranoside, methyl 2,3,4-tri-*O*-methyl-; Octyl (E)-2-methylbut-2-enoate; Butanoic acid, 3-hydroxy-, ethyl ester; 2,4-Decadienoic acid, ethyl ester, (E,Z)-; 4H-Pyran-4-one, 3,5-dihydroxy-2-methyl-; Methyl 4-(prop-1-en-2-yl)cyclohex-1-enecarboxylate. This cluster is characterized by relatively stable abundance at DAF40, followed by a sharp increase from DAF70 to DAF100, indicating that this period represents the peak biosynthetic phase for these key flavor and metabolic compounds.

In the present study, (E)-2-hexen-1-ol acetate is a C6 ester derived from the LOX pathway was among the esters that accumulated sharply in ‘Queen Nina’ from DAF70 to DAF100 (Cluster 5, Fig. 2F). This increase is consistent with the role of this compound as a transitional aroma marker bridging “fruity” notes, as previously reported in wine grape varieties including ‘Cabernet Sauvignon’ and ‘Shiraz’, where its concentration doubles after véraison (Qian et al., 2016). However, in contrast to these wine grape varieties, the accumulation of (E)-2-hexen-1-ol acetate in ‘Queen Nina’ was accompanied by a broader range of esters,particularly hydroxy esters such as butanoic acid, 3-hydroxy-, ethyl ester and pentanoic acid, 3-hydroxy-, ethyl ester. These hydroxy esters, which impart intense “fruity” aromas (Fenoll et al., 2009), showed a marked increase during late ripening. This broad accumulation of diverse esters underscores the importance of ester biosynthesis as the primary driver of the characteristic “fruity” aroma of mature ‘Queen Nina’ grapes.

Similarly, medium-chain unsaturated fatty acid ethyl esters such as ethyl (Z)-4-decenoate and ethyl (E,Z)-2,4-decadienoate typically exhibit “fatty”, “waxy”, and “citrus-like fruity” aromas. These compounds are directly derived from the esterification of fatty acids, and they are frequently associated with “dairy” or “tropical fruit” related aroma (Šikuten et al., 2025).

(2) Cluster 9 exhibited a continuous increase throughout the ripening period and comprised n-propyl acetate; and 2-ethoxy-2-methylpropane. n-Propyl acetate, as a short-chain fatty acid ester, may be generated during the DAF100 due to the general increase in ethanol concentration within the berry, which facilitates esterification with short-chain acids such as propanoic acid, catalyzed by alcohol acyltransferases (AATs). Lee et al. reported a similar accumulation pattern of propyl acetate during the ripening stage of ‘Muscadine’ grapes (Lee et al., 2016). In ‘Queen Nina’, this continuous accumulation further supports the progressive activation of ester biosynthesis during ripening, contributing to the overall “fruity” aroma of mature berries.

### 3.5 Key aroma contributors revealed by ROAV and sensory descriptors

**Fig. 3:**
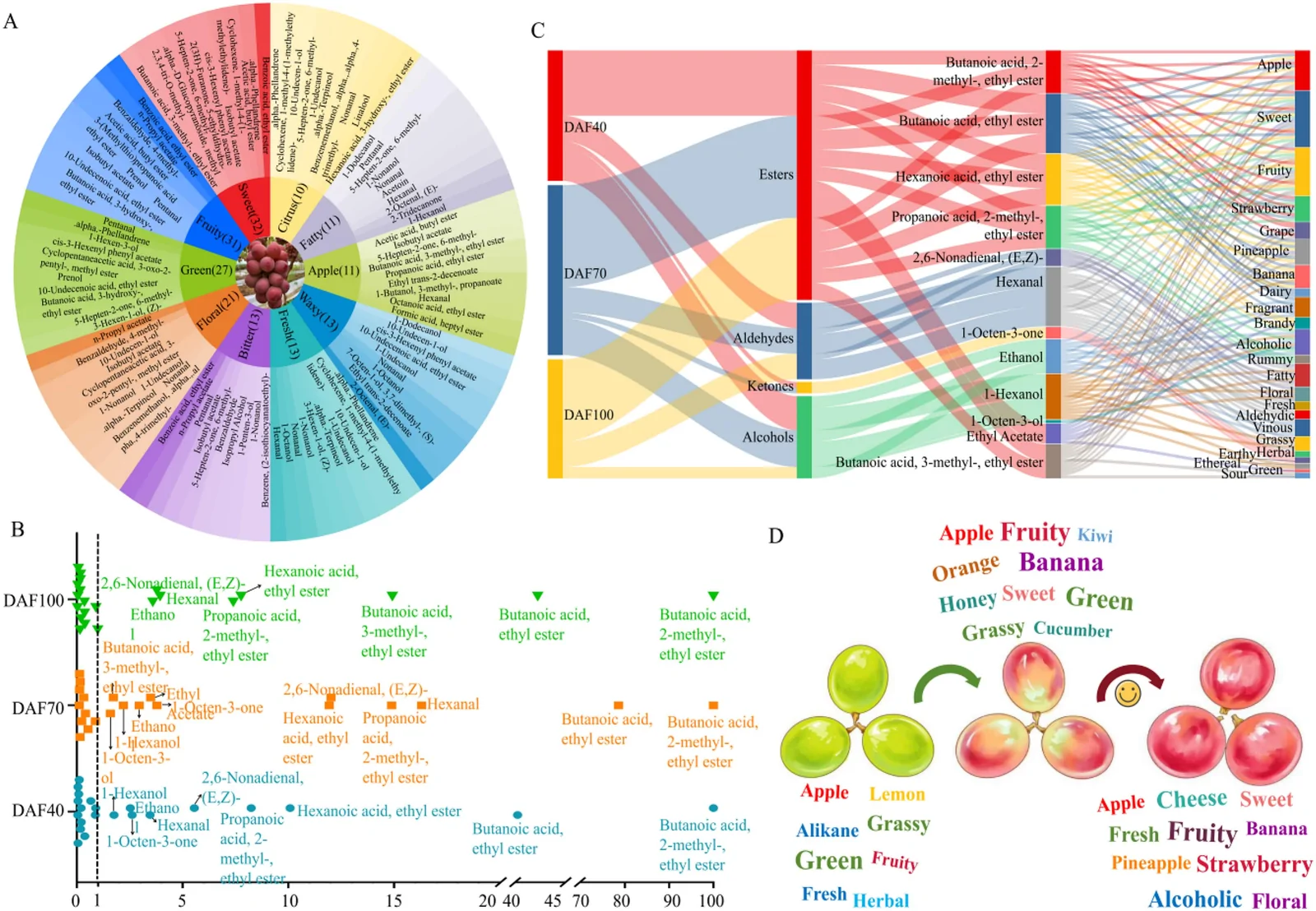
Relative odor activity value (ROAV) analysis and aroma profile characterization of ‘Queen Nina’ grapes at different developmental stage. (A) Flavor wheel illustrating the distribution of 81 aroma-active compounds across ten primary sensory descriptors. (B) ROAV analysis of the contribution of VOCs to the aroma of ‘Queen Nina’ grapes at DAF40, DAF70, and DAF100. Compounds with ROAV >0.1 are shown, and those with ROAV >1 at each respective stage are labeled. (C) Alluvial diagram illustrating the flow of aroma contributions: from developmental stage (left) to compound classes, then to specific VOCs, and finally to their corresponding aroma descriptors (right). (D) Aroma descriptor profiles of ‘Queen Nina’ at DAF40, DAF70, and DAF100. Font size of each descriptor represents the frequency of VOCs contributing to that aroma note at the respective stage.

The contribution of aroma-active compounds to grape flavor depends not only on their concentrations but also on their odor thresholds. To further characterize the contribution of VOCs to the overall grape aroma, ROAV were evaluated in this study. Typically, an ROAV >1 indicates that a volatile compound plays a dominant role in the overall aroma profile; compounds with ROAV values between 0.1 and 1 are regarded as exerting a moderate influence, while those with values <0.1 contribute minimally (Zhao et al., 2025). Among the 81 aroma-active compounds detected (Table S3; Fig. 3A), 22 exhibited ROAV >0.1 and were selected for further analysis (Fig. 3C). These compounds are likely key contributors to the distinct flavor profiles of ‘Queen Nina’ grapes across developmental stages (Fig. 3B). Based on compounds with ROAV >1 at the DAF100 stage, a characteristic flavor Sankey diagram was constructed, delineating key aroma attributes including “apple”, “pineapple”, “brandy”, “fruity”, “alcoholic”, “buttery”, “cheese”, and “fatty” (Fig. 3D).

The ester profile of ‘Queen Nina’ grapes in this study differed markedly from that previously reported by Wang, Zhang, et al. (2025), who identified five compounds (phenylacetaldehyde, 2,5-dimethyl-4-hydroxy-3(2H)-furanone, ethyl octanoate, ethyl crotonate, and ethyl (E,Z)-2,4-dodecadienoate) as maturity markers in the same cultivar. In contrast, our samples from Binchuan County were dominated by short- to medium-chain esters particularly ethyl butanoate, ethyl hexanoate, and their branched-chain derivatives, with “butanoic acid, 3-methyl-, ethyl ester” showing the most pronounced increase during ripening (ROAV: 0.31 → 14.92). Notably, butanoic acid, 2-methyl-, ethyl ester and butanoic acid, ethyl ester maintained exceptionally high ROAV values (>40) throughout all developmental stages, indicating their constitutive importance to the aroma of this cultivar. This compositional divergence likely reflects the influence of geographical and climatic factors: Binchuan County is characterized by high solar radiation and large diurnal temperature variations, conditions known to promote ester biosynthesis in grape berries (Farris et al., 2025). The synergistic combination of these esters each contributing overlapping “pineapple”, “creamy”, and “cheese” notes appears to underpin the characteristic creamy mouthfeel of mature ‘Queen Nina’ grapes, consistent with the additive effects of multiple esters observed in other fruits (Gonda et al., 2010).

Studies have confirmed that most ‘Queen Nina’ exhibit pronounced vinous aroma characteristics (Fei et al., 2026). This study revealed that esters in ‘Queen Nina’, such as Butanoic acid, ethyl ester and Hexanoic acid, ethyl ester typically exhibit “fruity” and “sweet wine” characteristics. Meanwhile, alcohols and aldehydes, including hexanal and 1-octen-3-ol, may contribute to the complex “brandy” aroma. The synergistic presence of these components may collectively shape the distinctive vinous aroma characteristic of ‘Queen Nina’,distinguishing it from terpenoid- or norisoprenoid-driven vinous profiles in other varieties.

### 3.6 Biomimetic sensory characterization of aroma and taste

**Fig. 4:**
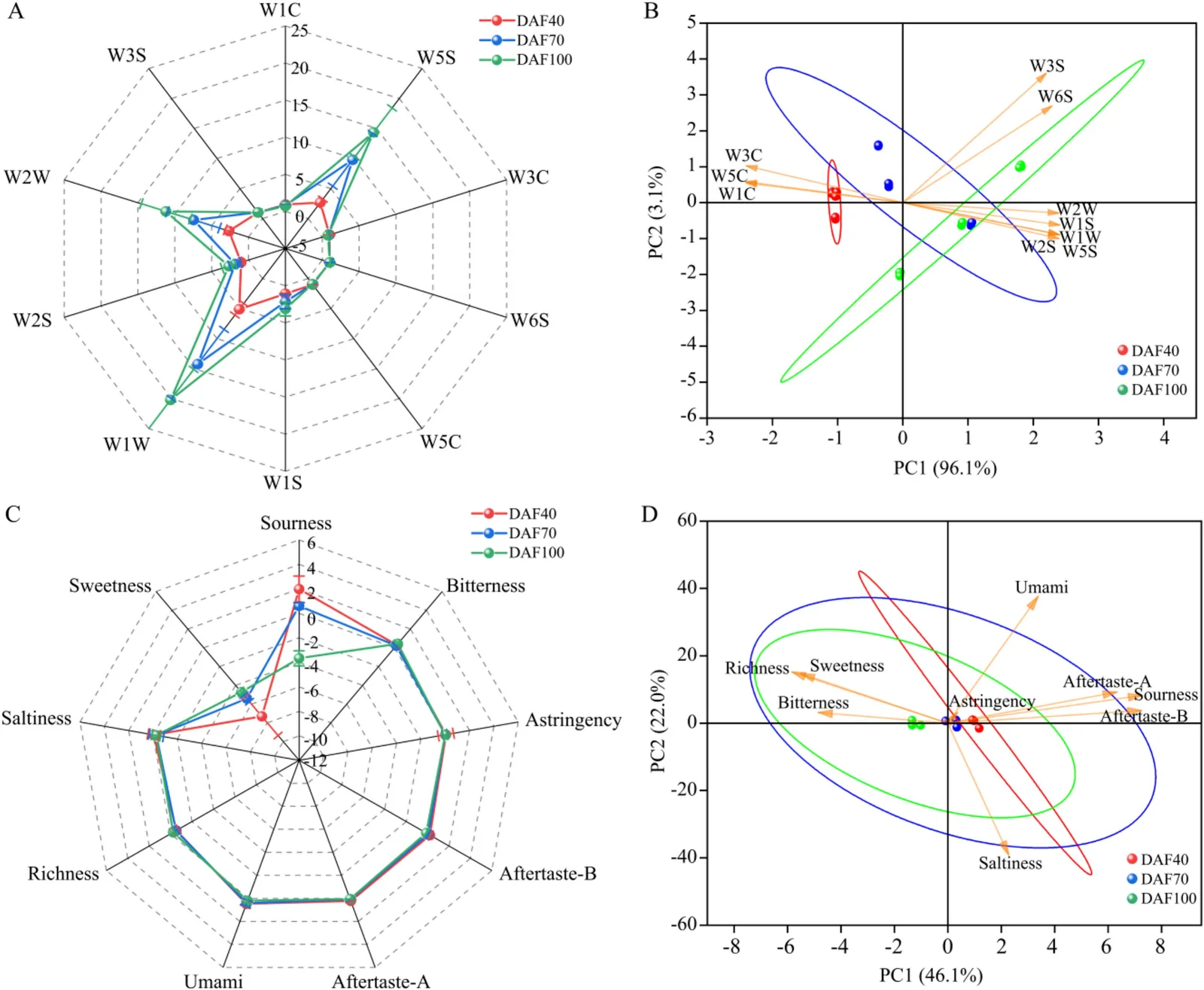
Electronic sensory analysis of ‘Queen Nina’ grapes at three developmental stage (DAF40, DAF70, and DAF100). (A) Principal component analysis (PCA) of electronic nose data. (B) Radar chart of electronic nose responses. (C) Principal component analysis (PCA) of electronic tongue data. (D) Radar chart of electronic tongue responses. For PCA plots, each point represents an individual sample, and the confidence ellipses indicate the 95% confidence region for each stage.

Electronic nose (E-nose) technology simulates the human olfactory system to conduct objective and quantitative assessments of the overall aroma profile of fruits using multi-sensor arrays. Principal component analysis (PCA) was performed on the E-nose response data of ‘Queen Nina’ collected at different developmental stages. The results showed that samples from distinct developmental phases exhibited clear separation in the PCA score plot (Fig. 4A). The contribution rate of PC1 reached 96.1%, while that of PC2 was 3.1%, yielding a cumulative contribution rate of 99.2%. This high cumulative variance indicates that the first two principal components adequately characterized the overall aroma differences among the samples.

Radar chart analysis further revealed the dynamic patterns of specific aroma components (Fig. 4B). Sensors with relatively high response values included W1W, W2W, W5S, W2S, W3S, and W1C, suggesting that the aroma profile of ‘Queen Nina’ is primarily characterized by sulfur-containing compounds, aromatic components, organic sulfides, broad-range volatiles (including alcohols and alkanes), and small-molecule nitrogen oxides. This pattern is broadly consistent with the importance of sulfur-containing and aromatic compounds in grape varietal aroma (Kalua & Boss, 2009; Qian et al., 2016). However, the particularly high responses of W1W and W2W (sulfur-selective sensors) in ‘Queen Nina’ are noteworthy, as they suggest a greater contribution of sulfur volatiles to the aroma profile of this cultivar compared to varieties where these sensors show lower relative responses. Notably, the responses of W1W, W2W, and W5S increased progressively during ripening and were markedly enhanced at DAF100. This trend likely reflects the accumulation of sulfur-containing volatiles and aromatic compounds during late maturation, consistent with the increased abundance of esters and other secondary metabolites observed in our GC–MS analysis (Fig. 2). Although sulfur compounds are often trace components, their low odor thresholds contribute substantially to the aroma complexity of mature ‘Queen Nina’ grapes.

In contrast, the sensors W3S, W6S, W1C, W5C, and W3C—which are sensitive to alkanes, hydrogen, and aromatic compounds showed relatively stable responses throughout ripening, suggesting that these volatile classes are not major drivers of the developmental shift in ‘Queen Nina’ aroma. This stability is consistent with previous reports that certain terpenoids and other volatiles remain at consistent levels during grape ripening (Wang, Yan, et al., 2025). Finally, these result suggests that the characteristic aroma arises from the interplay of ester-driven fruitiness and sulfur-contributed complexity.

In addition to aroma, taste is a critical factor influencing consumer preference. The electronic tongue (E-tongue) simulates human taste perception using artificial lipid membrane sensor arrays. PCA was performed on the E-tongue response data of ‘Queen Nina’ grapes at different developmental stages, revealing distinct separation among samples from various developmental phases (Fig. 4C). PC1 accounted for 46.1% of the total variance, and PC2 for 22.0%, yielding a cumulative contribution rate of 68.1%. This indicates that the first two principal components effectively distinguished the taste profiles across the developmental stages.

The clear separation of DAF100 samples from DAF40 and DAF70 samples along the PC1 axis confirmed significant differences in taste characteristics between mature grapes and those in earlier developmental stage. As shown in Fig. 4D, the responses for the five basic tastes (sour, sweet, bitter, salty, and umami) as well as astringency varied significantly across the developmental stages. VIP analysis identified Sourness, Aftertaste-A (astringency), and Umami as the key discriminators (VIP > 1) distinguishing the flavor profiles. Specifically, DAF40 samples were primarily characterized by high Sourness, Aftertaste-B (bitterness), and Aftertaste-A. DAF70 samples tended to cluster with Umami, Astringency, and Saltiness. This taste transition is consistent with the typical sugar-acid rebalancing during grape maturation, where accumulating sugars mask acidity. The high Sweetness and Richness scores at DAF100 suggest that ‘Queen Nina’ develops a balanced sweet-umami profile that may distinguish it from sourness- or bitterness-dominated varieties, where “fruity” aroma compounds enhance the perception of sweetness (Small & Prescott, 2005).

### 3.7 Untargeted metabolomics and characterization of core differential non-volatile metabolites

**Fig. 5:**
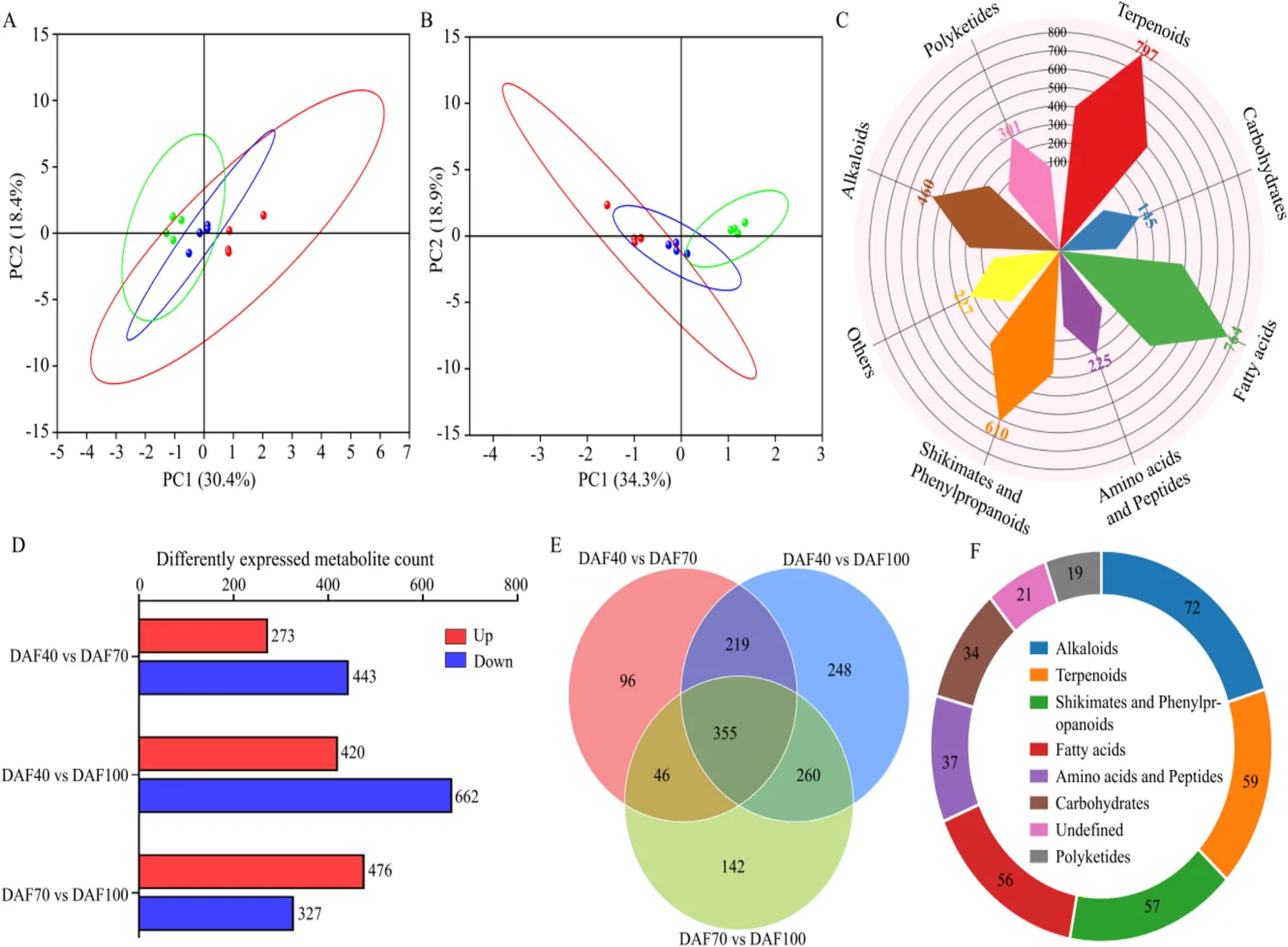
Non-volatile metabolomic profiling of ‘Queen Nina’ grapes during ripening. (A, B) PCA score plots of metabolite profiles in positive (A) and negative (B) ion modes at DAF40, DAF70, and DAF100. (C) Rose plot showing the compositional classes of total metabolites (3529 compounds). (D) Numbers of differentially accumulated metabolites in pairwise comparisons. (E) Venn diagram of differential metabolites among the three comparisons. (F) Donut chart depicting the classification and counts of the 355 shared differential metabolites. (For interpretation of the references to color in this figure legend, the reader is referred to the web version of this article.)

Untargeted metabolomics revealed significant metabolic reprogramming during ‘Queen Nina’ ripening. PCA clearly separated samples from the three developmental stages (Fig. 5A, B). A total of 3529 non-volatile metabolites (NVMs) were detected, including 797 terpenoids, 764 fatty acids, 610 shikimic acid/phenylalanine derivatives, 460 alkaloids, 301 polyketides, 225 amino acids and peptides, 145 carbohydrates, and 227 other compounds (Fig. 5C; Table S4). The OPLS-DA score plots showed distinct clustering of samples from different stages, and permutation tests confirmed the robustness of the models without overfitting (Fig.S2A-C). Pairwise comparisons (VIP > 1, *P* < 0.05) identified 716, 803, and 1082 differential metabolites between DAF40 vs. DAF70, DAF70 vs. DAF100, and DAF40 vs. DAF100, respectively, with up/down-regulated ratios of 273/443, 476/327, and 420/662 (Fig. 5D; Fig. S2D–F).

To identify core markers consistently driving metabolic changes, a Venn diagram across the three comparisons yielded 355 common differential NVMs (Fig. 5E). These metabolites represent the strict intersection of compounds that met the criteria of VIP > 1 and *P* < 0.05 in all three pairwise comparisons, ensuring their consistent alteration during the developmental process. These were classified into eight classes: alkaloids (72), terpenoids (59), shikimic acid/phenylpropanoid derivatives (57), fatty acids (56), amino acids/peptides (37), carbohydrates (34), undefined compounds (21), and polyketides (19) (Fig. 5F; Table S5).

#### 3.7.1 NVMs of carbohydrates

**Fig. 6:**
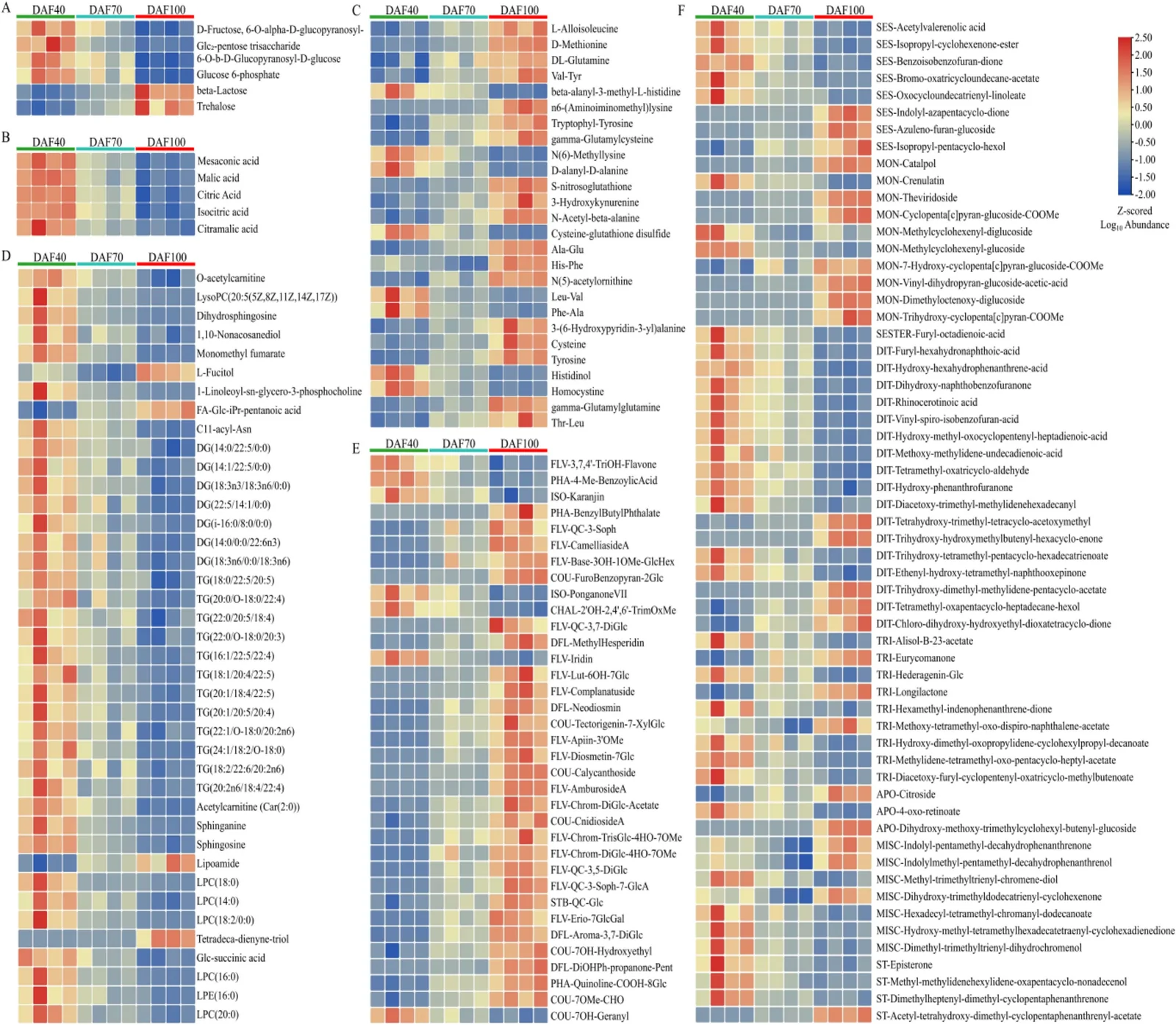
Heatmap visualization of dynamic changes in key non-volatile differential metabolites (NVMs) across six major classes during ‘Queen Nina’ grape ripening (DAF40, DAF70, and DAF100). (A) Carbohydrates; (B) Organic acids; (C) Amino acids and their derivatives; (D) Fatty acids and lipid derivatives; (E) Polyphenols (including flavonoids, phenolic acids, coumarins, isoflavones, and stilbenes); (F) Terpenoids (including monoterpenes, sesquiterpenes, diterpenes, triterpenes, and sesterterpenes). Color scales represent relative abundance (red indicates up-regulation, blue indicates down-regulation). Compound labels include prefixes denoting structural subclasses: flavonoids (FLV-), dihydroflavonoids (DFL-), chalcones (CHAL-), isoflavones (ISO-), coumarins (COU-), phenolic acids (PHA-), stilbenes (STB-); and terpenoids: monoterpenes (MON-), sesquiterpenes (SES-), sesterterpenes (SESTER-), diterpenes (DIT-), triterpenes (TRI-), apocarotenoids (APO-), miscellaneous (MISC-), and steroids (ST-). (For interpretation of the references to color in this figure legend, the reader is referred to the web version of this article.)

A total of 14 carbohydrates were identified as differential metabolites during growth, and the accumulation of these saccharides may directly contribute to the significant increase in total soluble sugar content and perceived sweetness during the post-ripening process of the berries (Fig. 6A). The accumulation of soluble sugars in grape primarily relies on the starch-sugar pathway (Bao et al., 2026). At DAF40, the content of d-fructose, 6-O-α-d-glucopyranosyl-, 6-O-β-d-glucopyranosyl-d-glucose and glucose 6-phosphate continuously increases, indicating the activation of active sugar metabolic pathways. The subsequent decline in these metabolites during the DAF100 may signify the conversion of sugars into their final storage forms, reflecting the metabolic characteristics of berries entering a stable maturation phase. Glycolysis and the pentose phosphate pathway are active the ripening stage is characterized predominantly by the accumulation of hexoses (Colantonio et al., 2022).

#### 3.7.2 NVMs of organic acids

Organic acids are critical determinants of grape flavor and quality, playing an important role in maintaining the balance of fruit acidity, energy metabolism, and the supply of precursors for secondary metabolism (Chen, Mkunga, et al., 2025). During the development of ‘Queen Nina’, organic acid metabolism exhibited pronounced stage-specific characteristics. Mesaconic acid, maleic acid, malic acid, citric acid, and isocitric acid showed a sharp accumulation at DAF40, followed by a continuous decline from DAF70 to DAF100 (Fig. 6B).

The development of sour taste in berries involves the synthesis of organic acids, primarily malic acid and citric acid, which are stored in large quantities within the vacuoles. After véraison, malic acid stored in the vacuoles is released into the mitochondria to enter the TCA cycle for energy supply; concurrently, a portion is decarboxylated via malic enzyme to form pyruvate. Following véraison, these acids undergo rapid degradation due to shifts in respiratory metabolism and dilution effects, a pattern that aligns strongly with the physiological phenomenon of continuously decreasing titratable acidity (TA) and increasing pH during grape ripening (Etienne et al., 2013).

#### 3.7.3 NVMs of amino acids

A total of 35 differential metabolites were identified as amino acids and their derivatives in ‘Queen Nina’. K-means clustering classified these 35 amino acid-related metabolites, with 21 of them being up-regulated during the ripening process. These included two essential amino acids (Histidinol and L-Alloisoleucine) that were abundantly produced during the DAF100. Additionally, three non-essential amino acids (DL-Glutamine, Tyrosine, and Homocystine) and 16 amino acid derivatives exhibited a positive correlation with growth. Moreover, beta-alanyl-3-methyl-L-histidine, N(6)-methyllysine, D-alanyl-D-alanine, and cysteine-glutathione disulfide exhibited high abundance during the DAF100. Furthermore, five compounds, including D-methionine, N6-(aminoiminomethyl)lysine, S-nitrosoglutathione, cysteine, and gamma-glutamylglutamine, were consistently down-regulated throughout the entire ripening process (Fig. 6C).

In this study, sulfur-containing amino acids including cysteine, glutathione, and their derivatives were abundant during the DAF40 but showed a consistent decline toward maturity (Fig. 6C). This pattern is consistent with the well-established role of sulfur assimilation in early fruit development, where cysteine and glutathione function as key antioxidants to mitigate oxidative stress during rapid cell expansion (Amiri et al., 2024). The early accumulation of glutathione also enables the generation of γ-glutamyl peptides, which may contribute to amino acid transport and secondary metabolism regulation. The subsequent decline of these sulfur-containing compounds during late ripening likely reflects a metabolic shift: as the need for antioxidant defense diminishes, sulfur and nitrogen resources are reallocated toward the biosynthesis of ripening-associated metabolites, including volatile thiols and esters.

The detection of compounds such as 4-hydroxybenzylamine and N(6)-methyllysine also suggests that the polyamine metabolic pathway is activated during early developmental stage. Polyamines are well-established growth regulators involved in cell division, development, and abiotic stress responses (Amiri et al., 2024). Their early activation in ‘Queen Nina’ may reflect a physiological response to the oxidative stress associated with rapid cell expansion during early berry growth. Notably, during the transition to ripening, polyamine-related compounds can serve as precursors for volatile thiols and as methyl donors for the modification of ripening-associated genes, thereby contributing to the formation of characteristic grape aroma (Dudareva et al., 2013).

#### 3.7.4 NVMs of fatty acids

A total of 40 fatty acids and their derivatives were identified as differential metabolites. These lipids were primarily categorized into 19 glycerides, 8 glycerophospholipids, 3 sphingolipids, 3 fatty acid esters, 3 fatty acyls, and 4 fatty amides and fatty glycosides. These include a large number of lysophosphatidylethanolamines (LPE), lysophosphatidylcholines (LPC), triglycerides (TG), and other phospholipid molecules. As degradation products of PC and PE, apart from LPE (16,0), compounds such as LysoPC (20:5(5Z,8Z,11Z,14Z,17Z)), LPC (18,0/0,0), and most LPCs were up-regulated during the DAF100 (Fig. 6D).

Most triacylglycerols (TGs) and diacylglycerols (DGs) were markedly down-regulated during ripening (Fig. 6D), consistent with the well-established role of these neutral lipids as energy reserves that are hydrolyzed to provide carbon skeletons and energy for sugar accumulation and secondary metabolism (Colantonio et al., 2022). The onset of véraison appears to trigger this lipolytic process, releasing free fatty acids that serve as direct precursors for ester biosynthesis, this finding supported by the concurrent accumulation of fatty acid-derived esters in Cluster 5 (Fig. 2F). Additionally, the conversion of TGs and DGs into membrane lipid components (e.g., phosphatidylcholine) via diacylglycerol kinase pathways releases free choline, which may contribute to osmotic adjustment and oxidative stress tolerance during ripening (Bao et al., 2026). The pronounced down-regulation of TGs/DGs in ‘Queen Nina’ highlights the active mobilization of lipid reserves to support ester biosynthesis during ripening, suggesting that lipid metabolism may be particularly active in this tetraploid table grape.

The decrease in TG/DG during ripening coincided with a marked increase in lysophosphatidylcholines (LPCs) including LysoPC(20,5), LPC (18,0/0,0), LPC (16,0), and LPC (20,0) and free choline at DAF100 (Fig. 6D). This concurrent shift is consistent with the conversion of TGs and DGs into membrane lipid components such as phosphatidylcholine via diacylglycerol kinase pathways, releasing free choline that may function as an osmoprotectant and antioxidant precursor (Bao et al., 2026).

Furthermore, during the growth and developmental stage of ‘Queen Nina’, the levels of four alcohols (Dihydrosphingosine, L-Fucitol, Sph(d18:0), and Sphingosine) were found to increase. These alcohols are subsequently converted into esters by alcohol acyl-CoA transferase (Podolyan et al., 2010), providing an additional precursor pool for the ester biosynthesis that characterizes the “fruity” aroma of mature ‘Queen Nina’ grapes (Cluster 5, Fig. 2F).Thus, fatty acid metabolism in ‘Queen Nina’ serves dual roles: supporting membrane remodeling and stress adaptation while providing precursors for the ester biosynthesis that underpins its characteristic “fruity” aroma.

#### 3.7.5 NVMs of polyphenols

Polyphenols are important secondary metabolites involved in the phenylpropanoid pathway in grape. They not only exert a decisive influence on fruit coloration, flavor characteristics, and aging potential but also endow the fruit with remarkable bioactivities, including antioxidant, anti-inflammatory, and cardiovascular protective effects (Han et al., 2025; Kalua & Boss, 2009).

We revealed that the polyphenolic compounds that changed during ‘Queen Nina’ ripening were classified into five classes: flavonoids, phenolic acids, coumarins, isoflavones, and stilbenes (Fig. 6E). Flavonoids were the most prominent, with the majority showing significant up-regulation at DAF100. Among these, Two anthocyanin components 6-Hydroxyluteolin 7-glucoside and Diosmetin 7-O-beta-D-glucopyranoside accumulated markedly during the DAF100, consistent with the established role of anthocyanins as the primary pigments responsible for the purplish-red coloration of red grapes (Li et al., 2025). Coumarins are a class of bioactive compounds with positive effects such as antimicrobial, anticancer, antiviral, and antioxidant activities (Bao et al., 2026). In this study, a total of six coumarins were identified to be abundantly produced during the DAF100 of grapes.The concurrent accumulation of these compound classes highlights the metabolic integration of color, flavor, and bioactivity in mature ‘Queen Nina’ grapes.

Phenolic acid compounds are important phenylpropanoid metabolites in grape berries. High concentrations of phenolic acids provide antioxidant protection for young tissues (Wang et al., 2023). In this study, 4-methylbenzoic acid was abundant at DAF40 but declined during ripening (Fig. 6E). This early-to-late decline contrasts with the sustained accumulation of flavonoid and anthocyanin, suggesting a division of labor among phenolic classes: phenolic acids for early defense, flavonoids for late coloration and antioxidant capacity. Furthermore, phenolic acids can serve as copigments, engaging in molecular stacking interactions with anthocyanins to enhance the color intensity of red wines and delay browning(Li et al., 2025).

#### 3.7.6 NVMs of terpenoids

Terpenoids are composed of isoprene units and, based on their carbon skeleton size, can be classified into monoterpenes, sesquiterpenes, diterpenes, triterpenes, and tetraterpenes. Throughout the grape lifecycle, they fulfill multiple functions ranging from fundamental physiological processes to ecological adaptation (Li et al., 2025). A total of 45 terpenoid compounds were identified in ‘Queen Nina’, comprising 17 diterpenes, 10 monoterpenes, 8 sesquiterpenes, 9 triterpenes, and 1 sesterterpene (Fig. 6F).

During the grape DAF40, monoterpenes and diterpenes exhibited a trend of first increasing and then decreasing. This pattern may be attributed to the abundant synthesis of most monoterpenes in grapes, where they impart intense floral aromas (e.g., in Muscat-type varieties) to attract pollinators such as bees. In contrast, another class of terpenoids, primarily sesquiterpenes and triterpenes, was substantially up-regulated during the DAF100. The triterpenes accumulated in the berry skin are involved in the formation of the cuticular wax, building a physical barrier to reduce water loss and resist pathogen attachment, whereas the sesquiterpenes serve as major contributors to woody and spicy aroma notes (Yue et al., 2020).

### 3.8 Integrated metabolic pathways and regulatory mechanisms underlying flavor formation

**Fig. 7:**
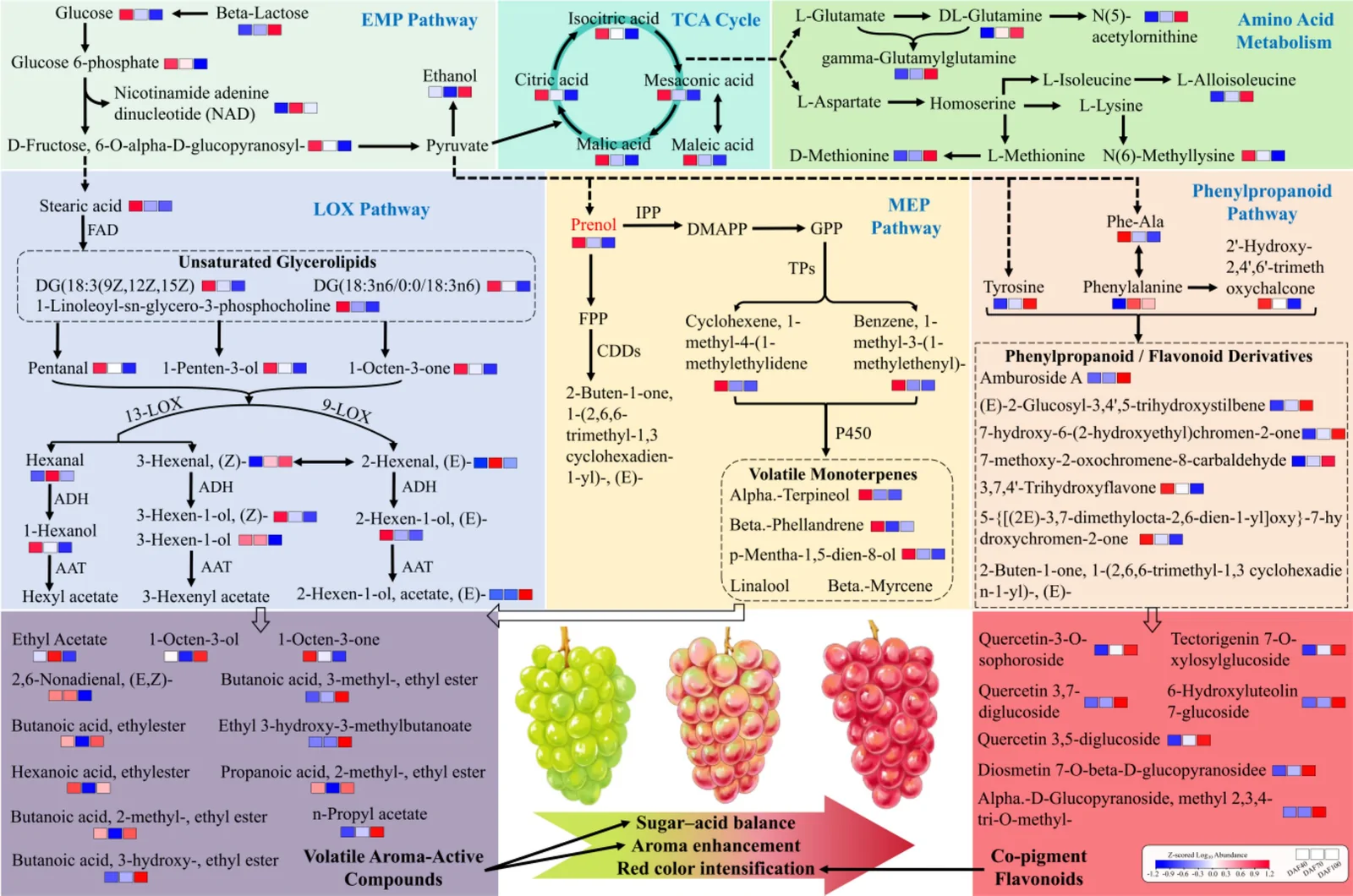
Schematic representation of the metabolic mechanisms underlying flavor and quality formation in ‘Queen Nina’ grapes across three developmental stage (DAF 40, 70, and 100). The map illustrates the convergence of glycolysis, the TCA cycle, amino acid metabolism, the LOX pathway, the MEP pathway, and phenylpropanoid biosynthesis. Color gradients and arrow directions indicate the temporal dynamics of metabolite accumulation (red: up-regulated at DAF100; blue: down-regulated). Abbreviations: EMP, Embden–Meyerhof–Parnas; TCA, tricarboxylic acid; LOX, lipoxygenase; MEP, methylerythritol phosphate; AAT, alcohol acyltransferase. (For interpretation of the references to color in this figure legend, the reader is referred to the web version of this article.)

The transition from early development (DAF40) to full ripening (DAF100) in ‘Queen Nina’ was characterized by a progressive decline in ethers, alcohols, and aldehydes—compounds enriched in Cluster 3 (Fig. 2F) and a concomitant sharp increase in esters, predominantly represented by Cluster 5 compounds (Fig. 2F). This indicates a fundamental metabolic shift from LOX-derived green volatiles to ester-dominated “fruity” aromas during ripening. The biosynthetic networks underlying this transition encompass multiple interconnected pathways, including fatty acid metabolism (providing precursors for ester synthesis), amino acid catabolism (supplying branched-chain esters and sulfur-containing volatiles), terpene biosynthesis (contributing floral notes), and monosaccharide/glycoside metabolism (providing carbon skeletons and glycosylation capacity). The coordinated activation of these pathways during late ripening in ‘Queen Nina’ highlights the metabolic integration that drives the characteristic aroma profile of this cultivar. In summar, the integration of key differential metabolites data enabled the construction of a comprehensive metabolic pathway map delineating flavor evolution during ‘Queen Nina’ grape ripening (Fig. 7).

Amino acids such as valine, leucine, isoleucine, alanine, and cysteine serve as direct precursors of aroma compounds in fruits, and their metabolic processes generate substantial amounts of alcohols, aldehydes, esters, and carbonyl compounds. Phenylalanine, on the other hand, is a precursor of phenolic substances, which constitute the foundation of the basic “fruity” aroma of grapes (Li et al., 2023; Villano et al., 2023). Monosaccharides not only contribute to sweetness but also serve as carbon skeletons for aromatic compound synthesis; intermediates derived from the TCA cycle can feed into the MEP and MVA pathways for terpene biosynthesis (Deluc et al., 2007). While the marked degradation of TGs and DGs during late ripening (Fig. 6D) may release fatty acid precursors that potentially support the abundant ester synthesis observed in Cluster 5 (Fig. 2F). Flavonol and flavone glycosides, which accumulated at DAF100 (Fig. 6E), function not only as non-volatile pigments but also as metabolic intermediates linking primary and secondary metabolism. These coordinated pathway illustrate how primary metabolism fuels flavor and color development in ‘Queen Nina.

In the present study, the progressive decline of LOX-derived C6 aldehydes and alcohols represented by Cluster 3 compounds including (Z)-3-hexen-1-ol and pentanal (Fig. 2F) provides direct evidence for the decreasing activity of the LOX pathway during ripening. This decline is consistent with the established LOX-HPL-ADH-AAT cascade, in which polyunsaturated fatty acids are sequentially converted to C6 aldehydes (via LOX and HPL), then to C6 alcohols (via ADH), and finally to esters (via AAT) (Podolyan et al., 2010; Qian et al., 2016). The decline in LOX-derived green volatiles (GLVs) during late ripening in ‘Queen Nina’ mirrors the pattern observed in wine grape varieties such as ‘Cabernet Sauvignon’, where unsaturated C6 aldehyde concentrations are significantly lower post-veraison compared to the growth stage (Kalua & Boss, 2009). However, the persistence of detectable levels of (Z)-3-hexen-1-ol until DAF100 in ‘Queen Nina’ suggests a more gradual decline than that reported in some wine grape varieties, potentially contributing to the complexity of the mature aroma profile. This shift from GLVs to esters marks the fundamental transition from ‘green’ to “fruity” aroma profiles in ‘Queen Nina’, and is consistent with the concurrent accumulation of esters observed in Cluster 5 (Fig. 2F).

Furthermore, monoterpenes and their derivatives including terpinen-4-ol and α-terpineol were abundant at DAF40 but declined sharply by DAF70 (Cluster 8, Fig. 2F), indicating that terpenoid metabolism in ‘Queen Nina’ is primarily active during early development rather than contributing directly to the mature aroma profile. This pattern contrasts with muscat-type varieties, where monoterpenes accumulate throughout ripening and serve as key contributors to floral aromas via the MEP pathway within the plastidial (Piombino et al., 2010; Šikuten et al., 2025). In ‘Queen Nina’, the early accumulation of these compounds may reflect their role as precursors for glycosidically bound forms a storage mechanism mediated by UDP-glycosyltransferases (UGTs) rather than as free volatile contributors at maturity (Fei et al., 2026). This interpretation is consistent with the rapid decline of free monoterpenes after DAF70, which may represent their conversion into non-volatile glycoside conjugates. Additionally, the limited contribution of terpenoids to the mature aroma of ‘Queen Nina’ is further supported by the dominance of esters (Cluster 5, Fig. 2F) during late ripening. While CCD-mediated cleavage of carotenoids generates norisoprenoids that contribute to “fruity” aroma in some grape varieties (Liu et al., 2025), their role in ‘Queen Nina’ appears secondary to ester biosynthesis. Collectively, these findings suggest that in ‘Queen Nina’, terpenoid metabolism is largely confined to early development, with glycosylation serving as a key regulatory mechanism for controlling the pool of free aroma compounds, while ester biosynthesis dominates the mature flavor profile.

## 4 Conclusion

This study systematically characterized the dynamic changes in volatile and non-volatile metabolites during the ripening of ‘Queen Nina’ table grapes through an integrated volatilomics, metabolomics, and electronic sensory approach. A total of 44 core differential VOCs governing the transition from “grassy” to “fruity” and “cheesy”aroma were identified. Among these, the progressive decline of (Z)-3-hexen-1-ol and pentanal marked the loss of green aroma, whereas the sharp accumulation of esters including 2-Hexen-1-ol, acetate, (*E*)-, 3-hydroxy-, ethyl ester, n-Propyl acetate and various medium-chain fatty acid ethyl esters contributed to the characteristic “fruity”, “cheesy”, and “tropical” aromas. ROAV analysis further pinpointed Butanoic acid, ethyl ester, Hexanoic acid, ethyl ester, Ethyl Acetate and their branched-chain with ROAV values exceeding 40 throughout all developmental stages. Furthermore, glycolysis and TCA cycle intermediates were active in early stages, while the downregulation of sulfur-containing amino acids and the degradation of lipids may potentially supply precursors for volatile biosynthesis. Concurrently, the upregulation of flavonoids and anthocyanins at DAF100 enhanced fruit coloration and antioxidant capacity.

In summary, this study demonstrates the metabolic basis of the distinctive aroma characteristics of ‘Queen Nina’ grapes. These findings provide a basis for further investigation into the quality characteristics and formation mechanisms of this cultivar. It should be noted that this study was conducted on a single cultivar (‘Queen Nina’) and therefore does not include direct comparison with contrasting cultivars exhibiting different aroma characteristics. Future research employing aroma recombination and omission experiments, as well as comparative analyses across multiple cultivars with contrasting flavor profiles, will be essential to definitively establish the causal relationships between specific volatile compounds and the perceived aroma characteristics of ‘Queen Nina’ grapes.

## CRediT authorship contribution statement

**Qiuping Li:** Writing – original draft, Investigation, Formal analysis, Data curation. **Zilin Wang:** Investigation, Formal analysis, Data curation. **Bo Zhang:** Investigation, Data curation. **Yameng Liu:** Investigation, Data curation. **Min Yang:** Writing – review & editing, Supervision, Resources, Funding acquisition. **Xinyi Huang:** Supervision, Project administration, Funding acquisition, Conceptualization.

## Funding

This work was supported by the “Science and Technology Talent Project of the Three Districts in Yunnan Province” (300220250000075), the Yunnan Provincial “Xingdian” Talents Support Program (No.YDYC-CYCX-2025-0009) and the “Nanjing Program of Yunnan Minzu University”.

## Declaration of competing interest

The authors declare that they have no known competing financial interests or personal relationships that could have appeared to influence the work reported in this paper.

## Data Availability

Data will be made available on request.
