# Supplementary material for: Integration of volatilomics, non-targeted metabolomics, and electronic sensory technologies reveals the flavor evolution mechanism in ‘Queen Nina’ table grapes during ripening
Source: Food Chem X. 2026 Sep 12;39:104436. doi: 10.1016/j.fochx.2026.104436 (PMC13594597; doi:10.1016/j.fochx.2026.104436)
Supplement: Supplementary file 1 [file mmc1.docx]

**Fig. S1.** Orthogonal partial least squares discriminant analysis (OPLS-DA) and volcano plots of differential volatile organic compounds (VOCs) in ‘Queen Nina’ grapes at different developmental stages. (A–C) OPLS-DA score scatter plots (left) and corresponding permutation test plots (right) for pairwise comparisons: (A) DAF40 vs DAF70, (B) DAF40 vs DAF100, and (C) DAF70 vs DAF100. (D–F) Volcano plots showing differentially accumulated VOCs in pairwise comparisons: (D) DAF40 vs DAF70, (E) DAF40 vs DAF100, and (F) DAF70 vs DAF100.

**Fig. S2.** Orthogonal partial least squares discriminant analysis (OPLS-DA) and volcano plots of non-volatile differential metabolites in ‘Queen Nina’ grapes at different developmental stages. (A–C) OPLS-DA score scatter plots (left) and corresponding permutation test plots (right) for pairwise comparisons: (A) DAF40 vs DAF70, (B) DAF40 vs DAF100, and (C) DAF70 vs DAF100. (D–F) Volcano plots showing differentially accumulated metabolites in pairwise comparisons: (D) DAF40 vs DAF70, (E) DAF40 vs DAF100, and (F) DAF70 vs DAF100.
